# The Right Stuff: On the Future of Nanotoxicology

**DOI:** 10.3389/ftox.2019.00001

**Published:** 2019-11-26

**Authors:** Bengt Fadeel

**Affiliations:** Nanosafety and Nanomedicine Laboratory, Institute of Environmental Medicine, Karolinska Institutet, Stockholm, Sweden

**Keywords:** microbiome, nanomaterial, nanomedicine, systems biology, toxicology

George Whitesides remarked, in his excellent perspective on the “right” size in nanobiotechnology, that “there already exists a highly developed science concerned with biologically relevant nanostructures: this science is called *chemistry*” (Whitesides, 2003). To this, one may add that there also exists a scientific discipline dealing with host responses to foreign objects on the nano- and microscale—it is called *immunology* (Shvedova et al., 2010). Whitesides goes on to explain that “biology also provides unparalleled examples of functional nanostructures to excite the imagination of nanotechnologists of all persuasions” (Whitesides, 2003). Indeed, as pointed out by Bruce Alberts in another visionary perspective, “the entire cell can be viewed as a factory that contains an elaborate network of interlocking assembly lines, each of which is composed of a set of large protein machines” (Alberts, 1998). These protein “machines” are oftentimes of nanoscale dimensions (van den Heuvel and Dekker, 2007).

Nanomedicine holds tremendous promise, yet despite the huge number of basic and preclinical studies, few nanomedicines have reached the clinic (Chan, 2017). Perhaps we have underestimated the complexity of biological systems, and that of human disease? Perhaps, as pointed out in a recent review, we need to view organs and cells in the body as complicit in the actions of nanomedicines: the chemistry of a material is altered upon contact with a biological system, and these changes determine its fate and function in the body (Chan, 2017). The reciprocal nature of so-called nano-bio interactions could be exploited for therapeutic gain if the underlying mechanisms are understood. However, the same features that may prove useful in the context of a disease may also turn out to be involved in the unwanted effects of a nanomaterial; toxicology and medicine may, in some respects, be viewed as two sides of the same coin.

## Expect the Unexpected

One of the frequently debated questions in the field is whether nanomaterials elicit “novel” or unanticipated effects not seen for the same material in its bulk form? Some experts have argued that this is not the case, and it has been suggested that “the focus on the search for ‘nano-specific’ [toxicities] may have the effect of ‘re-inventing the wheel’ of what is already known for conventional particles” (Donaldson and Poland, 2013). Indeed, the focus on “novelty” may end up obscuring some risks while exaggerating others (Maynard, 2014). Arguably, novelty is what makes nanomaterials so appealing in the first place. However, as toxicologists, we should not confuse novel applications (of a nanomaterial) with unexpected risks. Ultimately, the cellular and molecular processes that are triggered or perturbed by a nanomaterial (or by ions that are released by the material, or by biomolecules that adsorb to the material) are likely to be conserved and, therefore, well-understood, or should at least, in principle, be possible to understand. Some experts have argued that evidence for novel size-dependent properties, rather than particle size *per se*, should inform the definition of nanoparticles from an environmental, health and safety perspective (Auffan et al., 2009). However, if our understanding of biological systems is imperfect then our appreciation of the “toxic potential” of nanomaterials (Nel et al., 2006) will also be imperfect. Therefore, to understand nanomaterial effects on biological systems, and attendant risks to human health and the environment, we need to learn more about biological systems. Granted, we also need robust test methods with which to evaluate new materials, and automated high-throughput screening can be used to speed up the analysis (Feliu and Fadeel, 2010; Damoiseaux et al., 2011), but fundamentally what is needed is a deeper understanding of *biology*. This, therefore, is a key challenge as the field of nanotoxicology moves forward.

## Facing the Nano-Bio Interface

Mildred Dresselhaus, pioneering nanoscientist and champion of women in science, once said, “if you don't have material, you don't have an experiment.” In toxicology, this appears to be almost axiomatic: we need materials in order to test them with respect to their potential hazard—sometimes this is done after the fact, but safety evaluation should ideally take place in tandem with the development of new materials (cf. safe-by-design). In nanotoxicology, this is especially true—one cannot evaluate or draw conclusions regarding the toxicological or biological effects of a nanomaterial without knowing the material (Warheit, 2010). Nanotoxicology deals essentially with structure-activity relationships: we seek to pin-point the specific properties (or ensemble of properties, as they may be intertwined) that drive biological effects. However, one may ask whether toxicology is merely an ancillary science tasked with testing “stuff” that has been deliberately or unintentionally produced by others? I would argue that this is not the case. Toxicology deals with the cellular and molecular mechanisms that are triggered or perturbed by a toxicant, and such studies may therefore drive scientific discovery. Take the example of cell death research, which is intimately and obviously linked to toxicology (Orrenius et al., 2011; Andón and Fadeel, 2013). Nanotoxicological research, in turn, offers an opportunity to explore the interactions between engineered or incidental materials and biological systems at the nanoscale (Shvedova et al., 2010). This also has important ramifications for nanomedicine: understanding interactions at the nano-bio interface is a prerequisite for the safe and efficacious deployment of nanomaterials in patients (Wang et al., 2019).

## Toward A Predictive Toxicology

Despite more than 15 years' worth of nanotoxicological research, it can be argued that we still don't understand the underlying mechanisms of toxicity (Valsami-Jones and Lynch, 2015). Indeed, it has even been suggested that a good number of studies have arrived at erroneous conclusions, due in part to the fact that excessively high doses of nanomaterials have been applied, i.e., doses that are unrealistic from the point of view of occupational or environmental exposure (Krug, 2014). Meanwhile, we are faced with an onslaught of new nanomaterials and it is becoming more and more apparent that we cannot resort to traditional risk assessment approaches with their strong reliance on animal data. In fact, legal reforms now mandate that data generated by alternative testing strategies be considered before requiring testing in animals (Nel and Malloy, 2017). This is fully in line with the landmark report from the US National Academy of Sciences, “Toxicity Testing in the Twenty-First Century: A Vision and a Strategy,” in which the evolution of toxicology from a predominantly observational science based to a large extent on *in vivo* (animal) models to a *predictive* science focused on the inclusion of target-specific, mechanism-based observations of human *in vitro* (cellular) responses was emphasized, to assist in prediction of risk of chemicals to humans (Collins et al., 2008).

It has been suggested that nanoparticles combine the properties of solids (e.g., fluorescence in the case of quantum dots) with mobility, a property that is typically ascribed to molecules (Stark, 2011). Thus, as suggested in a very instructive and useful review by Stark (2011), the failure of early nanotoxicological studies may be due to the fact that not all concepts in chemical safety can be readily translated to nano-sized objects. Developing validated and interference-free *in vitro* tests for nanomaterials remains an important challenge in nanotoxicology (Hussain et al., 2015).

High-throughput screening (HTS) and high-content screening (HCS) approaches are gaining traction in nanotoxicology (Li et al., 2018). To this end, systematic modification of physicochemical properties of libraries of nanomaterials combined with computational analyses (modeling) of the data is required (Bai et al., 2017). Shaw et al. (2008) provided an early example of “multidimensional” *in vitro* screening of a panel of 50 nanoparticles of varying core compositions and surface modifications using several different cell types and assays, and the authors derived robust structure-activity relationships by using this approach. In a more recent study, an image based, automated HCS platform adapted for 3D cell models was developed (Cutrona and Simpson, 2019). Using carboxyl-modified polystyrene nanoparticles, the authors proceeded to conduct an RNAi-based screen and could identify molecular regulators of nanoparticle trafficking. The development of relevant and robust model systems amenable to HCS/HTS represents an important priority in nanotoxicology (Li et al., 2018).

Omics approaches, including “next generation” sequencing of populations of cells, or single-cell analyses, promise to shed light on the biological effects of different classes of nanomaterials (Paunovska et al., 2019). Additionally, multi-omics approaches, combining for instance transcriptomics and proteomics, may yield further insights into the complex cellular reactions to nanomaterials (Gallud et al., 2019). However, with big data comes big responsibilities, and toxicologists should work closely with statisticians and bioinformaticians, not only at the data analysis and visualization step, but also at the onset of the study, to ensure that the experimental design is appropriate for the question that is being addressed (Paunovska et al., 2019). It is also important to validate the omics data, in order to anchor the predictions in a relevant biological setting.

## Personalized (NANO) Toxicology

Mice are not men, yet animal models (of the human condition) remain a bedrock of biological and toxicological research. However, isogenic mice do not reflect the variation in toxicity responses that are seen in a genetically diverse human population. Clearly, there is a need for a “personalized” toxicology that takes into account the genetic susceptibility to toxicants. This can be accomplished by using panels of inbred mice, instead of a single mouse strain, or by using populations of outbred mice. In a seminal study, mice from 25 different inbred strains were exposed to silver nanoparticles and novel candidate genes associated with nanoparticle-induced lung inflammation were identified by using genome-wide association mapping (Scoville et al., 2017). More research is needed to shed light on interindividual variations in the susceptibility to nanomaterial exposure. Furthermore, infants are not adults, and substantial differences may occur, for instance, with respect to particle deposition in the lungs, as shown for iridium nanoparticles in a study in rats (Semmler-Behnke et al., 2012).

The realization that the gut microbiome influences human physiology represents “a paradigm shift in modern medicine” (Kundu et al., 2017). Microbial communities residing in our gut also participate in biotransformation of drugs and other xenobiotic compounds, and host-microbiome interactions therefore need to be considered in the study of toxicology and environmental health (Dietert and Silbergeld, 2015). The fact that the composition of the microbiome is dynamic and exhibits interindividual variations opens a new frontier in personalized medicine (and toxicology) and suggests a link between the environment and human physiology and disease: the microbiome as proxy for the exposome. In contrast to the host genome, the microbiome (our “second genome”) is readily modifiable (Zmora et al., 2016). Understanding host-microbiome interactions represents another challenge in nanotoxicology as we move forward.

## The Future of Nanotoxicology

Nanotoxicology is a relatively new discipline that embraces new developments in toxicology including the application of systems biology approaches with which to model and predict perturbations inflicted by nanomaterials in a living system (Fadeel, 2015; Fadeel et al., 2018). Most importantly, nanotoxicology can be viewed as a lesson in interdisciplinarity, and a comprehensive understanding of nano-bio interactions can only be obtained through a combination of different perspectives found in chemistry, physics, molecular biology, immunology, pharmacology, computational sciences, and so forth. Continuous communication across scientific disciplines is, therefore, critically important.

We must also acknowledge that the future of nanotoxicology lies in the hands of our students. Therefore, training the next generation of toxicologists to ensure that they are well-equipped in dealing with materials of ever increasing sophistication is of great importance. Indeed, as highlighted by Maynard et al. (2011) almost a decade ago, toxicology as a scientific discipline will need to move beyond “nano” and toward a toxicology of new and emerging materials. Here, nanotoxicology may provide a useful template.

## Author Contributions

BF handled the entirety of this paper.

### Conflict of Interest

The author declares that the research was conducted in the absence of any commercial or financial relationships that could be construed as a potential conflict of interest.

## References

[B1] Alberts B. (1998). The cell as a collection of protein machines: preparing the next generation of molecular biologists. Cell 92, 291–294. 10.1016/S0092-8674(00)80922-8 9476889

[B2] Andón F. T. Fadeel B. (2013). Programmed cell death: molecular mechanisms and implications for safety assessment of nanomaterials. Acc. Chem. Res. 46, 733–742. 10.1021/ar300020b 22720979

[B3] Auffan M. Rose J. Bottero J. Y. Lowry G. V. Jolivet J. P. Wiesner M. R. (2009). Towards a definition of inorganic nanoparticles from an environmental, health and safety perspective. Nat. Nanotechnol. 4, 634–641. 10.1038/nnano.2009.242 19809453

[B4] Bai X. Liu F. Liu Y. Li C. Wang S. Zhou H.. (2017). Toward a systematic exploration of nano-bio interactions. Toxicol. Appl. Pharmacol. 323:66–73. 10.1016/j.taap.2017.03.011 28344110 PMC5581002

[B5] Chan W. C. W. (2017). Nanomedicine 2.0. Acc. Chem. Res. 50, 627–632. 10.1021/acs.accounts.6b00629 28945418

[B6] Collins F. S. Gray G. M. Bucher J. R. (2008). Toxicology. Transforming environmental health protection. Science 319, 906–907. 10.1126/science.1154619 18276874 PMC2679521

[B7] Cutrona M. B. Simpson J. C. (2019). A high-throughput automated confocal microscopy platform for quantitative phenotyping of nanoparticle uptake and transport in spheroids. Small 15:e1902033. 10.1002/smll.201902033 31334922

[B8] Damoiseaux R. George S. Li M. Pokhrel S. Ji Z. France B.. (2011). No time to lose–high throughput screening to assess nanomaterial safety. Nanoscale 3, 1345–1360. 10.1039/c0nr00618a 21301704 PMC3980675

[B9] Dietert R. R. Silbergeld E. K. (2015). Biomarkers for the 21^st^ century: listening to the microbiome. Toxicol. Sci. 144, 208–216. 10.1093/toxsci/kfv013 25795652

[B10] Donaldson K. Poland C. A. (2013). Nanotoxicity: challenging the myth of nano-specific toxicity. Curr. Opin. Biotechnol. 24, 724–734. 10.1016/j.copbio.2013.05.003 23768801

[B11] Fadeel B. (2015). Systems biology in nanosafety research. Nanomedicine 10, 1039–1041. 10.2217/nnm.15.17 25929561

[B12] Fadeel B. Farcal L. Hardy B. Vázquez-Campos S. Hristozov D. Marcomini A.. (2018). Advanced tools for the safety assessment of nanomaterials. Nat. Nanotechnol. 13, 537–543. 10.1038/s41565-018-0185-0 29980781

[B13] Feliu N. Fadeel B. (2010). Nanotoxicology: no small matter. Nanoscale 2, 2514–2520. 10.1039/c0nr00535e 20877789

[B14] Gallud A. Klöditz K. Ytterberg J. Östberg N. Katayama S. Skoog T.. (2019). Cationic gold nanoparticles elicit mitochondrial dysfunction: a multi-omics study. Sci. Rep. 9:4366. 10.1038/s41598-019-40579-6 30867451 PMC6416392

[B15] Hussain S. M. Warheit D. B. Ng S. P. Comfort K. K. Grabinski C. M. Braydich-Stolle L. K. (2015). At the crossroads of nanotoxicology *in vitro*: past achievements and current challenges. Toxicol. Sci. 147, 5–16. 10.1093/toxsci/kfv106 26310852

[B16] Krug H. F. (2014). Nanosafety research–are we on the right track? Angew. Chem. Int. Ed Engl. 53, 12304–12319. 10.1002/anie.201403367 25302857

[B17] Kundu P. Blacher E. Elinav E. Pettersson S. (2017). Our gut microbiome: the evolving inner self. Cell 171, 1481–1493. 10.1016/j.cell.2017.11.024 29245010

[B18] Li Y. Wang J. Zhao F. Bai B. Nie G. Nel A. E.. (2018). Nanomaterial libraries and model organisms for rapid high-content analysis of nanosafety. Natl. Sci. Rev. 5, 365–388. 10.1093/nsr/nwx120

[B19] Maynard A. D. (2014). Is novelty overrated? Nat. Nanotechnol. 9, 409–410. 10.1038/nnano.2014.116 24894473

[B20] Maynard A. D. Warheit D. B. Philbert M. A. (2011). The new toxicology of sophisticated materials: nanotoxicology and beyond. Toxicol. Sci. 120(Suppl. 1):S109–S129. 10.1093/toxsci/kfq372 21177774 PMC3145386

[B21] Nel A. Xia T. Mädler L. Li N. (2006). Toxic potential of materials at the nanolevel. Science 311, 622–627. 10.1126/science.1114397 16456071

[B22] Nel A. E. Malloy T. F. (2017). Policy reforms to update chemical safety testing. Science 355, 1016–1018. 10.1126/science.aak9919 28280165

[B23] Orrenius S. Nicotera P. Zhivotovsky B. (2011). Cell death mechanisms and their implications in toxicology. Toxicol. Sci.. 119, 3–19. 10.1093/toxsci/kfq268 20829425

[B24] Paunovska K. Loughrey D. Sago C. D. Langer R. Dahlman J. E. (2019). Using large datasets to understand nanotechnology. Adv. Mater. 31:e1902798. 10.1002/adma.201902798 31429126 PMC6810779

[B25] Scoville D. K. Botta D. Galdanes K. Schmuck S. C. White C. C. Stapleton P. L.. (2017). Genetic determinants of susceptibility to silver nanoparticle-induced acute lung inflammation in mice. FASEB J. 31, 4600–4611. 10.1096/fj.201700187R 28716969 PMC5602892

[B26] Semmler-Behnke M. Kreyling W. G. Schulz H. Takenaka S. Butler J. P. Henry F. S.. (2012). Nanoparticle delivery in infant lungs. Proc. Natl. Acad. Sci. U.S.A. 109, 5092–5097. 10.1073/pnas.1119339109 22411799 PMC3323983

[B27] Shaw S. Y. Westly E. C. Pittet M. J. Subramanian A. Schreiber S. L. Weissleder R. (2008). Perturbational profiling of nanomaterial biologic activity. Proc. Natl. Acad. Sci. U.S.A. 105, 7387–7392. 10.1073/pnas.0802878105 18492802 PMC2396702

[B28] Shvedova A. A. Kagan V. E. Fadeel B. (2010). Close encounters of the small kind: adverse effects of man-made materials interfacing with the nano-cosmos of biological systems. Annu. Rev. Pharmacol. Toxicol. 50:63–88. 10.1146/annurev.pharmtox.010909.105819 20055698

[B29] Stark W. J. (2011). Nanoparticles in biological systems. Angew. Chem. Int. Ed Engl. 50, 1242–1258. 10.1002/anie.200906684 21290491

[B30] Valsami-Jones E. Lynch I. (2015). NANOSAFETY. How safe are nanomaterials? Science 350, 388–389. 10.1126/science.aad0768 26494749

[B31] van den Heuvel M. G. Dekker C. (2007). Motor proteins at work for nanotechnology. Science 317, 333–336. 10.1126/science.1139570 17641191

[B32] Wang Y. Cai R. Chen C. (2019). The nano-bio interactions of nanomedicines: understanding the biochemical driving forces and redox reactions. Acc. Chem. Res. 52, 1507–1518. 10.1021/acs.accounts.9b00126 31149804

[B33] Warheit D. B. (2010). Debunking some misconceptions about nanotoxicology. Nano Lett. 10, 4777–4782. 10.1021/nl103432w 21033694

[B34] Whitesides G. M. (2003). The 'right' size in nanobiotechnology. Nat. Biotechnol. 21, 1161–1165. 10.1038/nbt872 14520400

[B35] Zmora N. Zeevi D. Korem T. Segal E. Elinav E. (2016). Taking it personally: personalized utilization of the human microbiome in health and disease. Cell Host Microbe 19, 12–20. 10.1016/j.chom.2015.12.016 26764593

